# Optimization of Electrode Patterns for an ITO-Based Digital Microfluidic through the Finite Element Simulation

**DOI:** 10.3390/mi13101563

**Published:** 2022-09-21

**Authors:** Ze-Rui Song, Jin Zeng, Jia-Le Zhou, Bing-Yong Yan, Zhen Gu, Hui-Feng Wang

**Affiliations:** Key Laboratory of Smart Manufacturing in Energy Chemical Process Ministry of Education, East China University of Science and Technology, Shanghai 200237, China

**Keywords:** digital microfluidic, finite element simulation, electrowetting-on-dielectric, indium tin oxide, laser direct etching, droplet manipulation

## Abstract

Indium tin oxide (ITO)-based digital microfluidics (DMF) with unique optical and electrical properties are promising in the development of integrated, automatic and portable analytical systems. The fabrication technique using laser direct etching (LDE) on ITO glass has the advantages of being rapid, low cost and convenient. However, the fabrication resolution of LDE limits the minimum line width for patterns on ITO glasses, leading to a related wider lead wire for the actuating electrodes of DMF compared with photolithography. Therefore, the lead wire of electrodes could affect the droplet motion on the digital microfluidic chip due to the increased contact line with the droplet. Herein, we developed a finite element model of a DMF with improved efficiency to investigate the effect of the lead wire. An optimized electrode pattern was then designed based on a theoretical analysis and validated by a simulation, which significantly decreased the deformation of the droplets down to 0.012 mm. The performance of the optimized electrode was also verified in an experiment. The proposed simulation method could be further extended to other DMF systems or applications to provide an efficient approach for the design and optimization of DMF chips.

## 1. Introduction

Digital microfluidics (DMF) have been extensively studied for liquid handling at a microscale level [1]. Based on the effect of electrowetting-on-dielectric (EWOD), the DMF is able to control the wetting behavior of droplets through an array of electrodes and manipulate the droplets to split, dispense, mix and transfer without mechanical accessories [2,3,4]. Recent advances in manufacturing and electronic techniques have significantly promoted the development of DMF systems to achieve advantages such as a low power consumption, low reagent cost, miniaturization, flexibility and reconfiguration [5,6] Hence, the applications of DMF are increasingly being extended to fields such as point-of-care testing [7,8], cell research [9,10,11,12], biomedicine [13] and environmental monitoring [14,15].

Various techniques have been developed to manufacture DMF chips, including the photolithography process [16], printed circuit boards (PCBs) [17] and laser direct etching (LDE) on ITO glass [18]. Among these, LDE provides a low cost and rapid approach to fabricate the electrode array for ITO-based digital microfluidics (ITO-DMF) [19]. In practice, the processing time of LDE for a palm-size ITO-DMF chip may be no longer than a minute. The ITO-DMF also has the advantages of high transparency and a flat surface; thus, it is promising for most applications of microfluidics, especially for optical integrated analyses [20]. As it is difficult to fabricate a multilayer circuit on ITO glass, lead wires are required to enable an addressable voltage to be applied to each electrode in an ITO-DMF. However, the existence of lead wire can induce unwanted droplet deformation, causing a failure of the droplet control and an error in the droplet movement [21]. To address these issues, a decrease in the width of the lead wire can lower the deformation of the droplet, but the smallest width is limited by the laser spot size and etching process, which is approximately 80 μm using an LDE machine with an ultraviolet (UV) laser. Although the use of a femtosecond laser can provide a higher resolution down to the sub-micro level, it can spend hours fabricating a single chip and significantly increases the cost. Several related works have focused on developing an orifice-based ITO-DMF chip [22] and investigating the surface wettability and electrical resistance of ITO glass to improve the droplet control [23]. There is still a lack of studies focusing on droplet deformation induced by lead wire.

As the geometry of the electrodes affects the capillary force on the droplets according to the EWOD phenomenon, an alternative way to improve the control stability of an ITO-DMF is to modify the electrode pattern to compensate for the force induced by the lead wire. A similar strategy was used for the manipulation and distribution of droplets. For example, a heart-shaped electrode was developed for unidirectional droplet motion, making it possible to transfer droplets over a long distance with only two alternately applied actuation signals [24]. A method called “one to three” was developed for the generation of sub-microliter droplets by driving the mother droplets in two opposite directions on an embedded electrode [25]. A stripped electrode was designed for droplet splitting, which improved the efficiency and stability of a microdrop transport [26]. A jetting bar pattern was demonstrated to eject satellite droplets [27]. With electrode patterns of different sizes, a DMF system was developed for an automatic ELISA assay based on magnetic separation [28].

**Figure 1:**
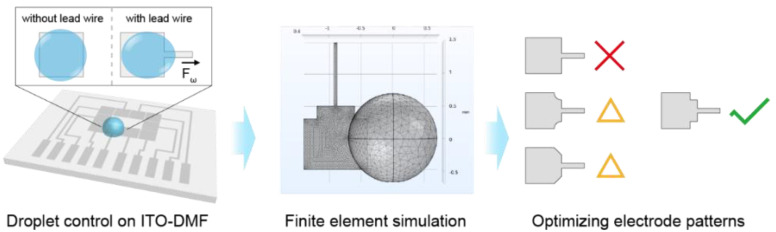
The force induced by the lead wire of electrodes on ITO-DMF causes deformation of the droplets and affects the droplet movement. A finite element model is developed for the optimization of the electrode pattern to enhance the stability of droplet control on ITO-DMF.

In this study, we aimed to reduce the influence of lead wire on the control stability of an ITO-DMF by optimizing the design of an electrode pattern with the assistance of a finite element simulation (Figure 1). A simplified DMF model was developed to investigate the effect of lead wire in the droplet control, through which the simulation time could be reduced by ~24%. According to the theoretical analysis, various electrode patterns were selected as candidates and tested through the proposed DMF model. An optimized pattern of the electrode was then obtained, which obviously decreased the deformation of the droplet induced by the lead wire. The result was also supported by the experiment results conducted on the ITO-DMF. It was also notable that the proposed method for electrode pattern optimization was not limited to the ITO-DMF; it could also be used for other DMF systems with different fabrication techniques to provide an efficient approach for the design and optimization of DMF chips.

## 2. Materials and Methods

### 2.1. Fabrication of the ITO-DMF Chip

An ITO-DMF chip was produced from commercial ITO glass with a thickness of 1.1 mm and a square resistance of ~8 Ω. Before etching, the ITO glass was ultrasonically washed with ethanol and ultrapure water for 3 min, respectively. A pulsed UV laser with a wavelength of 355 nm and a pulse duration of 20 μs was used to process the electrode pattern on the ITO glass. The ITO glass was then rinsed with ultrapure water to remove the residuals of the etching and dried by nitrogen. The electrode array on the ITO glass was tightly covered by a polytetrafluoroethylene (PTFE) film (10 μm, Hongfu Material, Dongguan, China), which worked as both dielectric and hydrophobic layers. To reduce the friction drag to the droplets, silicon oil (0.65 cs) was used to lubricate the PTFE film before the experiment.

### 2.2. Droplet Manipulation with the ITO-DMF Platform

A home-designed DMF system was used to manipulate the droplets on the ITO-DMF chip, which generated a high voltage with a charge pump circuit and modulated the voltage on each electrode through a multiplexing chip (HV507, Microchip Technology Inc., Chandler, AZ, USA) [29]. Droplet actuation was achieved by applying dc voltage (230 V). The device was mounted on a microscope (Nikon eclipse Ci, Tokyo, Japan) with a digital camera (E3ISPM08300KPC, ToupTek Photonics, Hangzhou, China) to monitor the droplet on the ITO-DMF chip. During the droplet manipulation, the deformation of the droplet was measured by using the camera software, which was calibrated by an objective micrometer.

### 2.3. Finite Element Simulation

**Figure 2:**
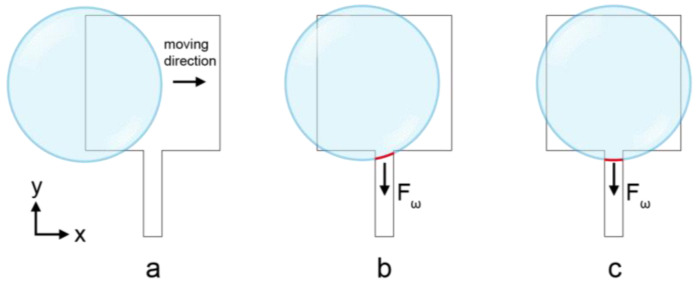
The simulation model for investigating the effect of lead wire on the deformation of a droplet. For the move-in model, a droplet driven by the capillary force toward the *x*-axis does not make contact with the lead wire at the initial stage (**a**); as it moves close to the center of the electrode, it makes contact with the lead wire and an additional force toward the direction of the lead wire is produced (**b**). For the activation model, the droplet is positioned at the center of the electrode and makes contact with the lead wire (**c**).

The models of the ITO electrodes with lead wires were built and simulated by COMSOL Multiphysics 6.0 software with the integration of the computational fluid dynamics (CFD) module, the single-phase and multiphase flow modules, the laminar flow module and the level set method. The model consisted of two phases of water and air. According to the theoretical analysis, the effect of the EWOD would result in the changing of the contact angle. In order to simplify the simulation process and lower the time consumption, we directly set the contact angle of the droplet with different surfaces to simulate the change in the contact angle caused by voltage in practice. The contact angle inside the activated electrode was set to 120° and the contact angle outside the electrode was set to 78°. The droplet was initialized as a hemisphere with a diameter of 1.4 mm and a volume of 0.72 μL. The length of the electrode side was 1 mm and the lead wire was connected to one side of the electrode at the center. A normal element size was used (except otherwise mentioned) to build the physics-controlled mesh as a compromise for accuracy and computing speed. A move-in model (Figure 2a,b) and an activation model (Figure 2c) with different initial states of droplets were developed to investigate the influence of lead wire on the droplet control. For the move-in model, the droplet was initially positioned on the edge of the activated electrode without a covering on the lead wire. For the activation model, the droplet was initially positioned in the center of the activated electrode with a covering on the lead wire. The simulations were run by a workstation with a Windows 10 operating system, 3.5 GHz running frequency and 16 GB random access memory.

## 3. Results and Discussion

### 3.1. Theoretical Analysis of the Electrode with Lead Wire

Typically, the electrode shape of an ITO-DMF is basically square. Each electrode is connected with an external control circuit via lead wire for the voltage application, which is also formed together with the electrodes by etching on the ITO glass. The width and direction of the lead wire are affected by the processing technique and layout of the DMF chip. A typical lead wire direction is perpendicular to the edge of the square electrode, as demonstrated in Figure 2a. When the width of the electrode is not much larger than the width of the lead wire, the effect of the wire on the movement of droplets cannot be ignored. In this case, the lead wire will act as part of the electrode. The liquid–gas contact angle of the droplets on the electrode where the electrical potential is applied satisfies the Young–Lippman equation [30]:

(1) $$cos\theta =cos\theta_{0}+\frac{C}{2\gamma }V^{2}$$

where θ is the contact angle with the electrical potential applied, $\theta_{0}$ is the zero potential contact angle, C is the capacitance of the electrode, γ is the liquid–gas interface surface tension and V is the applied voltage. When the droplet is on the electrode where the electric potential is applied, the contact angle of the droplet decreases according to the equation, leading to an unbalanced contact angle where part of the droplet is on the activated electrode and the other part of the droplet is out of the activated electrode. This unbalanced contact angle will produce an obvious wettability gradient and then generate a capillary force to drive the droplet deformation or movement, which satisfies the equation [31]:

(2) $$f_{\omega }=\gamma \left(cos\theta -cos\theta_{0}\right)$$

By integrating the above equation, the capillary force in a certain direction is obtained:

(3) $$F_{\omega }=L\gamma \left(cos\theta -cos\theta_{0}\right)$$

As the voltage on the electrode is applied through the lead wire, it is ineluctable that a part of the droplet on the hydrophobic interface remains at the hydrophilic interface, resulting in a larger contact length of the droplets in this direction. It is obvious that with an increase in the width of the lead wire, the deformation of the droplets in the direction of the lead wire will also increase.

### 3.2. Simulation of the Droplet Manipulation

**Figure 3:**
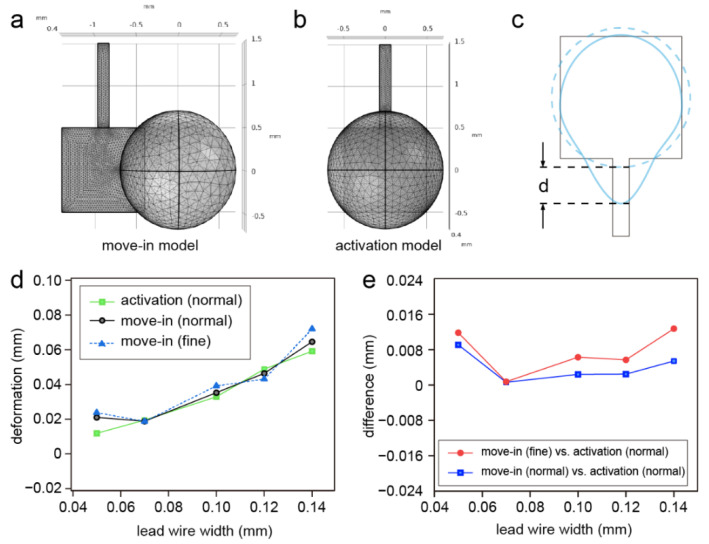
(**a**) The mesh of the move-in model with a normal element size; (**b**) the mesh of the activation model with a normal element size; (**c**) the definition of the droplet deformation; (**d**) simulation results of the droplet deformation based on the two models: the move-in model with a normal element size (black line) and a fine element size (dashed blue line) and the activation model with a normal element size (green line); (**e**) the difference in droplet deformations in comparison with the simulated activation model.

The simulation speed and accuracy of a finite element simulation can be affected by the mesh size and mesh density [32]. An increase in the grid density or the number of grid cells corresponding with a decrease in the element size will raise the computation time. Herein, to study the effect of lead wire on the droplet deformation, we first introduced the move-in model and the activation model for the simulation. The difference between the two models was the initial state of the droplet. For the move-in model, the droplet was initially located on the edge of the electrode and moved in to the center of the electrode due to the capillary force. For the activation model, the droplet was initially located in the center of the electrode. Both models were simulated with lead wire widths of 0.05, 0.07, 0.1, 0.12 and 0.14 mm; the degrees of freedom of the two models were approximately 200,000 (Figure 3a,b). We ran a grid independence test with the lead wire with a width of 0.05 mm and the deformation error between the different element sizes (normal and fine) was ~3.6%, which indicated that the configuration of the grid was suitable for the models. To evaluate the effect of the lead wire, we defined the deformation of the droplet as the extended length of the droplet induced by the lead wire (Figure 3c). As a result, the deformation of the droplets rose with an increase in the lead wire width in the activation model (Figure 3d), which was consistent with the theoretical analysis. However, we found an unexpectedly high deformation of the 0.05 mm lead wire in the move-in model when even a fine element size was used for the model. This unexpectedly high deformation with the 0.05 mm lead wire in the move-in model was mainly caused by the asymmetry of the mesh division as the capillary force induced by the mesh asymmetry was enhanced with the decrease in the lead wire width. Due to the symmetry of the mesh division, the capillary force induced by the asymmetric meshing was not obvious in the activation model. It was also notable that the difference between the move-in model and the activation model was not obvious; however, the computing time was reduced by ~24% with the activation model compared with the move-in model. When further increasing the lead wire width to 0.16 mm, the difference between the two models abruptly increased. Therefore, the activation model was suitable for predicting the performance of the electrodes when the lead wire width was no greater than 0.14 mm. 

### 3.3. Optimization of the Electrode Pattern

**Figure 4:**
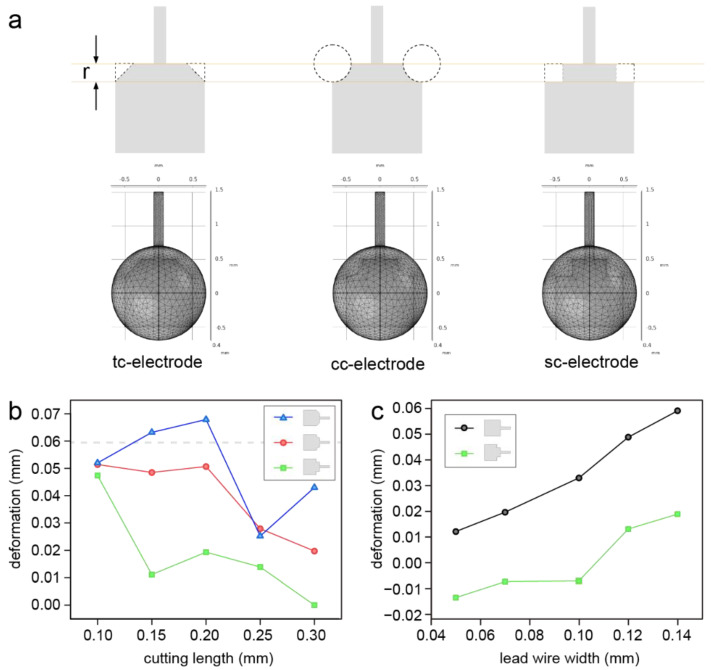
(**a**) The design of the three candidate electrodes and the corresponding simulation models; (**b**) The simulated droplet deformation on the three electrodes with cutting lengths ranging from 0.10 mm to 0.30 mm using the activation model. (**c**) The simulated droplet deformation on the square electrode and the sc-electrode with lead wire widths ranging from 0.05 mm to 0.14 mm.

According to the theoretical analysis, it could be inferred that the length of the contact line highly affected the deformation of the droplets. To address the deformation problem caused by the lead wire, we attempted to optimize the electrode pattern by reducing the effective contact line between the droplet and the electrode in the direction of the lead wire. We assumed that the cut part of the electrode corner at the same side of the lead wire could reduce the effective contact line and alleviate the droplet deformation. Three cutting shapes (triangle, circle and square) labelled as tc-electrode, cc-electrode and sc-electrode were selected as candidates and tested by a simulation based on the activation model (Figure 4a). The 0.14 mm lead wire was used in the simulation as its use is preferential in the LDE technique. In addition, the activation model was deemed suitable to be used to predict the performance of the electrode with a 0.14 mm width of lead wire, as discussed above. The results indicated that the usage of the cc-electrode and sc-electrode could significantly lower the deformation of the droplet (Figure 4b). When the cutting length of the modified electrode was only 0.1 mm, the droplet deformations were close to each other for the three electrodes and almost the same with the square electrode without optimization. The deformation then decreased with an increase in the cutting length of the sc-electrode and cc-electrode. The tc-electrode dramatically caused a higher deformation than the square electrode when the cutting length was no greater than 0.2 mm. As the sc-electrode had the best performance, the sc-electrode with a cutting length of 0.25 mm was tested with different lead wire widths ranging from 0.05 to 0.14 mm. As shown in Figure 4c, the sc-electrode could highly reduce the deformation in comparison with the square electrode. It was also notable that when the lead wire width was lower than 0.12 mm, a negative deformation was achieved by the sc-electrode, which meant that a higher force was provided opposite to the direction of the lead wire with this configuration. Therefore, the cutting length for the cutting part of an electrode should be set according to the lead wire width to avoid negative deformation.

### 3.4. Test of the Droplet Control on the ITO-DMF

**Figure 5:**
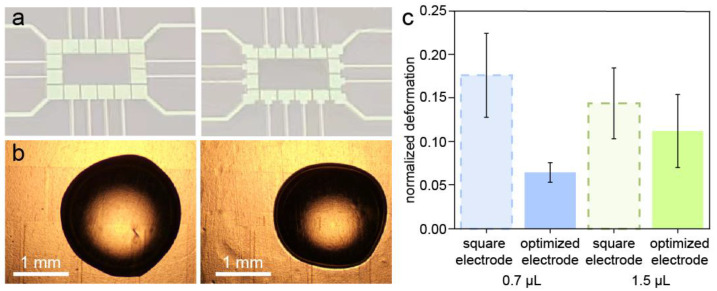
(**a**) Photos of the fabricated ITO-DMF chip with square electrode (left) and optimized electrode (sc-electrode, right); (**b**) the microscopic images of the droplet (1.5 μL) as actuated by the square electrode (left) and optimized electrode (right); (**c**) the normalized deformation of the droplets (0.7 μL and 1.5 μL) as measured based on the microscopic images (*N* = 10).

To examine the performance of the optimized electrode pattern in a real droplet control, ITO-DMF chips with a square electrode and an optimized electrode were fabricated and tested. As shown in Figure 5a, the side length of each electrode was 1 mm with a 0.14 mm width lead wire, which could be accurately processed by using laser etching. The gap between the electrodes was 65 μm. For the optimized electrodes, the corners at the two sides of the lead wire were etched to form a square with a 0.25 mm cutting length. A water droplet of 1.5 μL was loaded on the ITO-DMF chip and a voltage was applied to the chip to execute the droplet manipulation and the microscopy image was captured to measure the deformation by using the camera software. It was observed that the electrodes without optimization were more likely to cause a larger deformation of the droplets (Figure 5b). For example, the droplets could be deformed and move along with the lead wire, resulting in a bias from the center of the electrode and even movement out of the electrode region. The optimized electrode obviously decreased the deformation of the droplets, which is useful for the control stability of DMFs. In addition, the failure of the droplet control decreased with the optimized electrode; the observed existing failures were mainly caused by facts such as charge inverting, droplet evaporation and bubbles under the PTFE film. These issues were not related to the pattern of the electrode. The deformation of the droplets was measured based on the microscopic image when the voltage was applied to the electrode. To compare the deformation of the droplets with different volumes, we defined a normalized deformation as the ratio of the droplet deformation to the droplet diameter. As a result, the normalized deformation caused by the electrodes was lowered from 0.129 mm to 0.076 mm by using the optimized electrode compared with the square electrodes (Figure 5c), as predicted by the simulation. As a result, the normalized deformations of the 0.7 μL and 1.5 μL droplets were lowered from 0.1765 to 0.0649 and 0.1442 to 0.1124, respectively, which suggested that the degree of the optimization was related to the volume of the droplet. In addition, we calculated the average normalized deformation of the simulation with a droplet volume of 0.72 μL; the results before and after optimization were 0.048 and 0.0154, respectively. The difference between the results of the experiment and the simulation may have been caused by unpredictable conditions, including a charge accumulation in the droplet, the influence of oil on the contact angle and the unevenness of the hydrophobic surface.

## 4. Conclusions

In conclusion, we investigated the effect of lead wire on the droplet deformation of an ITO-DMF chip fabricated by LDE through a finite element simulation and an experiment. Two models (the move-in model and activation model) were developed for simulating the electrowetting process of the droplets. It was demonstrated that the two models had similar results with lead wire widths ranging from 0.07 mm to 0.14 mm; however, the activation model took less time and reduced the grid error for the simulation. Subsequently, the activation model was used for the optimization of the electrode patterns. Different electrode patterns were selected according to the theoretical analysis to reduce the effective contact line along with the lead wire. The simulation results demonstrated that the sc-electrode provided the lowest deformation (< 0.012 mm) among the electrodes with the same conditions. Furthermore, the simulation results were verified by an experiment. It was obvious that the use of the optimized electrode (sc-electrode) could lower the deformation of the droplet in comparison with a conventional square electrode. The optimized parameters of this study are applicable for certain configurations of ITO-DMFs such as the size of the electrode, the contact angle of the droplet and the electrowetting properties of the hydrophobic layer. This approach to electrode pattern modeling and optimization is flexible and convenient and may be used in various configurations. It can also be further extended and used in other types of DMFs for broad applications.
